# Digital pathways connecting social and biological factors to health outcomes and equity

**DOI:** 10.1038/s41746-025-01564-8

**Published:** 2025-03-20

**Authors:** Yan Cui

**Affiliations:** 1 Department of Genetics, Genomics and Informatics, University of Tennessee Health Science Center, Memphis, TN 38163 USA; 2 Center for Integrative and Translational Genomics, University of Tennessee Health Science Center, Memphis, TN 38163 USA; 3 Center for Cancer Research, University of Tennessee Health Science Center, Memphis, TN 38163 USA

**Keywords:** Medical ethics, Machine learning

## Abstract

Digital pathways extend conventional connections between social and biological factors and health outcomes, significantly influencing health equity. Data representation bias and distribution shifts are key mechanisms through which determinants of health impact generalizability of artificial intelligence (AI) models and subsequently affect health outcomes and equity. These mechanisms provide critical targets for algorithmic interventions, which can lead to Pareto improvements in AI model performance across diverse populations, thereby mitigating health disparities.

## Conventional pathways connecting social and biological factors to health outcomes

A wide range of social and biological factors affect health outcomes and disparities across diverse populations. The World Health Organization categorizes social determinants of health (SDOH) into structural and intermediary determinants^1^. Structural determinants, such as social class, gender, ethnicity, education, occupation, and income, create or reinforce social stratification and define individual socioeconomic positions. Intermediary determinants, such as living conditions, work environment, social and physical environments, and health behaviors, are influenced by structural determinants and connect to individuals’ immediate material and psychosocial circumstances. The structural determinants drive the differences in intermediary determinants, which in turn affect health outcomes and equity^1^. For instance, people with lower socioeconomic status often have less access to quality healthcare, which impacts the detection, treatment, and management of diseases.

Biological differences among populations, shaped by the history of human evolution, migration, and admixture, also have significant implications for health outcomes^2,3^. As human populations migrated and settled in diverse environments, they underwent genetic adaptations to local conditions, leading to differences in biological processes such as immune response and metabolism. Genomic studies have shown the impact of demographic phenomena, including migration, mixing, and population bottlenecks, on the evolutionary dynamics of disease-related genetic variations, influencing their distribution and prevalence^4^. For example, in the event of a population undergoing a bottleneck, the genetic diversity within the population decreases^5^, which may lead to the loss of specific alleles increasing the susceptibility to certain diseases, or conversely, the loss of alleles protecting against certain diseases.

Ultimately, all the contributing factors exert their influences on health outcomes by altering biological processes^6^. For instance, chronic stress associated with socioeconomic hardship or discrimination can induce physiological changes, such as increased inflammation and altered stress hormone regulation, contributing to conditions including hypertension, cardiovascular disease, diabetes, and mental health disorders. Additionally, socioeconomic status affects behaviors such as diet, physical activity, and alcohol and tobacco consumption. These behaviors can lead to various health conditions and contribute to biological differences, such as altered levels of cholesterol, blood pressure, and body mass index. Socioeconomic status also influences DNA methylation, a mechanism that modulates gene activity and expression, potentially affecting long-term health outcomes^7^.

## Emerging digital pathways influencing health outcomes

**Fig. 1:**
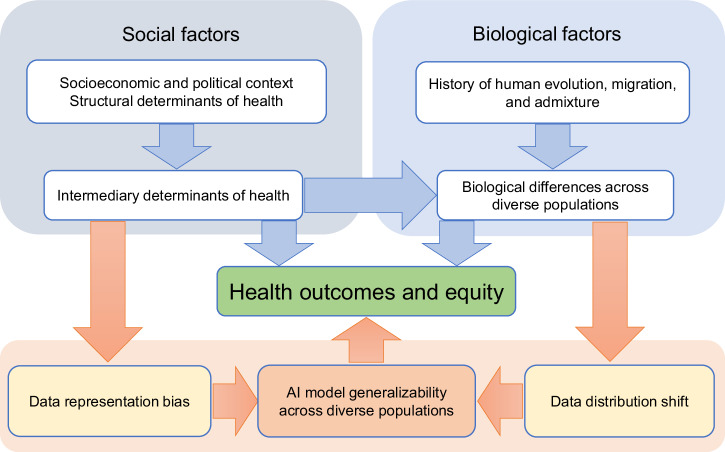
Conventional and digital pathways from social and biological factors to health outcomes. Data representation bias and distribution shifts are key mechanisms through which social and biological factors influence AI model performance across diverse populations, and subsequently, health outcomes and equity. The light blue and light orange arrows represent the conventional and digital pathways, respectively.

As clinical research and healthcare increasingly integrate big data and algorithms, digital pathways have emerged as critical extensions of the conventional pathways, creating additional routes and mechanisms for social and biological factors to influence health outcomes and equity (Fig. 1). Here, digital pathways refer to the processes by which data and algorithms convey and mediate the effects of social and biological determinants on health outcomes. For example, marginalized populations are less likely to participate in clinical trials due to mistrust or lack of access, which impacts health data collection and results in inadequate representation of these populations in health datasets^8–10^. Moreover, biological differences among populations can lead to distribution shifts in various types of data ranging from molecular to physiological levels, including genetic and somatic mutations^11^, epigenetic modifications^12^, RNA and protein expression^13^, metabolic profiles^14^, microbiome signatures^15^, reference intervals for clinical laboratory tests^16^, and disease risk^17^. Recent studies have shown that unequal representation and distribution shifts in health data significantly impact the generalizability and applicability of predictive models for clinical studies and healthcare, thus exacerbating health disparities^8,18–20^.

The digital pathways contain key targets for algorithmic interventions to promote health equity. Specifically, the impact of data representation bias and distribution shifts can be mitigated by using algorithms such as transfer learning. Transfer learning is a machine learning strategy that utilizes the knowledge and representations learned from a source domain with ample data to facilitate training a model for a target domain with sparse data^21^. For example, transfer learning has been used to address the impact of data representation bias and distribution shifts on clinico-genomic prediction of diseases such as Alzheimer’s disease and cancers^22^. Deep learning models were pretrained on data from a well-represented ancestry group (European) to capture general patterns, then fine-tuned on data from underrepresented ancestry groups (African American, East Asian, and Latin American). This process enables the model to adapt to the unique characteristics of the target domain, improving prediction accuracy across diverse populations. In principle, any population group with abundant data for a machine learning task can be used to enhance performance for another population group with limited data. A recent study, for example, used transfer learning to improve image-based prediction of refractive errors for South Asians by fine-tuning a model pretrained on a larger East Asian dataset^23^.

Current fairness-aware machine learning methods often involve a fairness-accuracy tradeoff, where achieving fairness may compromise prediction accuracy for certain population groups^24^. Transfer learning can avoid this tradeoff, potentially improving AI model performance for data-poor populations without compromising performance for data-rich populations and resulting in a Pareto improvement towards more equitable machine learning outcomes^22,25,26^. Pareto improvement is a concept from economics, denoting a change that benefits at least one party in the system without making any other party worse off^27^. In machine learning with diverse populations, a Pareto improvement indicates reducing model performance disparity without incurring the fairness-accuracy trade-off^22^.

SDOH data is essential for developing equitable machine learning models in healthcare^28–30^. However, existing SDOH datasets often have quality issues including implausibility and misclassification^31^. Low-quality SDOH data can introduce biases into machine learning models, potentially exacerbating health disparities^32^. Additionally, transfer learning carries the risk of propagating biases, as bias inheritance occurs when a pre-trained model passes biases from the source task to the target task, causing the target model to exhibit similar unfair patterns^33^. For example, large foundation models pre-trained on extensive yet biased datasets can transfer skewed representations to target models, impacting the fairness and reliability of their performance in downstream tasks^34^. Thus, it is important to debias machine learning models by removing or “unlearning” biased knowledge. Notably, a recent study suggests that unlearning biases in one field (e.g. gender) may also reduce biases in other fields (e.g. race and religion) in large language models (LLMs) due to shared underlying representations in the model’s embedding space^35^, potentially leading to more effective and comprehensive bias mitigation in LLMs.

In summary, representation biases and distribution shifts in health data, driven by social and biological factors, are key mechanisms in the emerging digital pathways that profoundly impact the generalizability and applicability of machine learning models across diverse populations. These mechanisms provide actionable targets for algorithmic interventions such as transfer learning, which can be applied to develop more equitable AI models and thus promote health equity.
